# Understanding the interaction between melatonin and bifidobacteria

**DOI:** 10.20517/mrr.2025.10

**Published:** 2025-05-15

**Authors:** Sonia Mirjam Rizzo, Giulia Longhi, Chiara Tarracchini, Chiara Argentini, Alice Viappiani, Massimiliano G. Bianchi, Ovidio Bussolati, Douwe van Sinderen, Marco Ventura, Francesca Turroni

**Affiliations:** ^1^Laboratory of Probiogenomics, Department of Chemistry, Life Sciences and Environmental Sustainability, University of Parma, Parma 43124, Italy.; ^2^GenProbio srl, Parma 43124, Italy.; ^3^Department of Medicine and Surgery, University of Parma, Parma 43125, Italy.; ^4^Interdepartmental Research Centre “Microbiome Research Hub”, University of Parma, Parma 43124, Italy.; ^5^APC Microbiome Institute and School of Microbiology, Bioscience Institute, National University of Ireland, Cork T12 YT20, Ireland.

**Keywords:** Melatonin, bifidobacteria, hormones, gut microbes, CST

## Abstract

**Aim:** The human gastrointestinal tract is home to a complex and dynamic microbial community, known as the gut microbiota, which begins to form at birth and evolves throughout life. Among the factors influencing its initial development, breastfeeding is one of the most important. Human milk is a chemically complex body fluid, including hormones, like melatonin, which is involved in regulating the sleep-wake cycle, helping to establish the newborn’s circadian rhythm. In the current study, the molecular interactions between human melatonin and a bifidobacteria-rich infant gut microbiota were explored.

**Methods:** Possible molecular communication was assessed using *in vitro* assays and functional genomic approaches.

**Results:** Our results highlight that melatonin elicits different functional microbial impacts, both at transcriptional and phenotypic levels (i.e., adhesion to intestinal cells), that are dependent on the bifidobacterial species analyzed.

**Conclusion:** Among the bifidobacterial taxa assayed, *Bifidobacterium bifidum* demonstrated the highest level of molecular interaction with melatonin, highlighting its significant role in this process.

## INTRODUCTION

The human gastrointestinal tract (GIT) is colonized by a complex microbial ecosystem, collectively referred to as the gut microbiota, whose establishment begins immediately after birth and evolves under the influence of multiple factors during the entire lifespan of an individual^[1-3]^. These include delivery mode (naturally or C-section), infant feeding (breastfeeding or formula), maternal health status, and environmental exposures^[4-6]^. Undoubtedly, among the factors that may affect colonization and development of the infant gut microbiota, breastfeeding is one of the most significant^[7-9]^. In this regard, human milk represents a nourishing body fluid rich in different components such as lactose, proteins, immunoglobulins, hormones, and human milk oligosaccharides (HMOs) that support the infant’s neurological development and shape the early immune system^[10-12]^. Besides its nutritional and immunological properties, human milk also contains a unique microbiome, comprising beneficial, commensal, and potentially probiotic bacteria, which are believed to contribute to the initial colonization of the infant gut^[13-15]^. Notably, among the first microbial colonizers of the neonatal gut, there are members of the genus *Bifidobacterium*, such as *Bifidobacterium bifidum*, which are proposed to be horizontally transmitted from the mother to the infant through breastfeeding^[16-19]^. These bacteria are particularly significant due to their crucial roles in enhancing immune function, protecting against pathogens, and maintaining gut barrier integrity^[20,21]^. While the effects of specific components of human milk - such as HMOs - on the gut microbiota and its metabolism have been well studied, the potential role of other elements like hormones is still largely unknown^[22,23]^. Among the hormones occurring in breast milk, melatonin has garnered increasing interest due to its pivotal role in infant physiology, particularly in regulating sleep^[24,25]^. This hormone is synthesized from tryptophan not only by the pineal gland but also by peripheral tissues, including gastrointestinal cells and mammary glands^[26,27]^. Since fetal melatonin production is initially absent, neonates rely on maternal breastfeeding as a source of this hormone^[28]^. Studies have shown that melatonin load in breast milk exhibits a diurnal variation, with significantly higher levels during the night, which may aid in regulating the infant’s sleep-wake cycle^[29]^. Moreover, melatonin levels decrease progressively throughout lactation, with the highest levels observed in colostrum^[30]^. While melatonin has been extensively studied for its role in sleep regulation and circadian biology^[31]^, its potential impact on the infant gut microbiota remains largely unexplored. In fact, only a few studies have investigated how melatonin may interact with gut-resident microbes. Available data suggest that this molecule can influence genes involved in biofilm formation, fimbria biogenesis, transcriptional regulation, carbohydrate transport and metabolism, phosphotransferase systems (PTS), stress response, and metal ion binding and transport. For instance, *Enterobacter aerogenes* exhibits increased motility and circadian-like activity in response to melatonin, an effect not observed in *Escherichia coli* or *Klebsiella pneumoniae*, indicating a species-specific response^[32,33]^. Additional evidence from perinatal rat models has shown that melatonin-treated gut microbiota promotes gut development by reducing oxidative stress, autophagy, and inflammation. These effects were associated with a higher abundance of beneficial SCFA-producing genera such as *Allobaculum*, *Bifidobacterium*, and *Faecalibaculum*, suggesting a mechanistic link between melatonin-modulated microbial communities and improved intestinal health^[34]^. These findings suggest a potential role for melatonin in modulating microbial behavior and host colonization. This knowledge gap is particularly relevant in the context of early-life microbial development, where bifidobacteria dominate the infant gut and rely heavily on milk-derived substrates^[4,23]^. Starting from this premise, here we evaluate the effect of melatonin on a typical infant microbiota dominated by bifidobacteria. By investigating how melatonin molecularly interacts with bifidobacterial cells, we sought to provide insights into the mechanism by which breast milk contributes to the establishment of healthy gut microbiota during infancy.

## MATERIALS AND METHODS

### Strains and cultivation conditions

*Bifidobacterium* strains used in this study were *B. bifidum* PRL2010, *Bifidobacterium breve* PRL2012, and *Bifidobacterium longum* subsp. *longum* PRL2022, previously identified as prototype strains for their respective species^[35,36]^. Strains of *Escherichia coli* 52F, *Enterococcus faecalis* 2F, and *Enterobacter hormaechei* 179F were isolated from infant fecal samples in our laboratory. Each bacterium was grown anaerobically in human colon environment-simulating growth medium (IGSM)^[37]^ supplemented with 0.05% (wt/vol) L-cysteine hydrochloride under anaerobic conditions (2.99% H_2_, 17.01% CO_2_, and 80% N_2_). The IGSM includes several types of carbohydrates, i.e., mucin (3 g/L) and starch (3 g/L), inulin (0.2 g/L), pectin (0.2 g/L), arabinogalactan (0.2 g/L), xylan (0.2 g/L), and lactose (0.2 g/L).

### ICST-BI/EN and *B. bifidum* PRL2010 exposure to melatonin

For the experiment, we selected a common infant community state type (I-CST) dominated by bifidobacteria and with the presence of other species (ICST-BI/EN). All bacterial strains were grown overnight from glycerol stocks in IGSM broth. Cells were then inoculated in 30 mL of freshly prepared IGSM broth supplemented with 2 μM (wt/vol) of melatonin^[38]^. Specifically, a Thoma cell counting chamber (Herka) was used to enumerate bacterial cells. Bifidobacterial cells were inoculated at a level of approximately 1 × 10^7^ cells/mL, while other strains at a level of around 1 × 10^6^ cells/mL. The cell load for each bacterial strain was determined based on both the individual growth rate of each bacterium and the actual physiological concentration observed in the I-CST^[39]^. Growth was observed until the exponential phase (approximately 1 × 10^8^ cells/mL), at which point cells were harvested by centrifugation at 6,000 rpm for 5 min. The bacterial cells were then stored at -80 °C until they were processed for RNA extraction. The same procedure was applied for *B. bifidum* PRL2010 when tested as a single culture.

### Effects of melatonin on bifidobacterial growth

To evaluate the melatonin susceptibility of *B. bifidum* PRL2010, the strain was grown in the presence of 18 different concentrations of melatonin using the broth microdilution method. Specifically, starting from a level of 8 μM melatonin, a 2-fold dilution series was achieved until reaching a concentration of 58.59 pM of melatonin and aliquoted in a 96-well microtiter plate. Subsequently, an overnight culture of *B. bifidum* PRL2010 cultivated in De Man-Rogosa and Sharpe broth (MRS) was diluted to get an optical density (OD_600nm_) of ~1 at 600 nm, and 15 μL of such a diluted cell suspension was used for inoculation of 135 μL MRS broth added with a specific melatonin concentration. The microtiter plates were incubated anaerobically at 37 °C for 48 h. A plate reader (BioTek, Winooski, VT, USA) was used to measure the optical densities at a wavelength of 600 nm. The OD_600nm_ values were measured in intermittent mode, with readings detected at 3-minute intervals three times for 48 h of growth. Each reading was preceded by 30 s of shaking at medium speed. The growth of the cultures is presented as the means of these replicates.

### Adhesion of bacteria to HT29-MTX cells

Bifidobacterial adhesion to HT29-MTX cells was evaluated according to the protocol outlined by Serafini *et al.*^[40,41]^. Briefly, human colorectal adenocarcinoma HT29-MTX cells (kindly furnished by Professor A. Baldi, University of Milan) were cultivated in Dulbecco’s modified Eagle’s medium (DMEM) with 10% fetal bovine serum (FBS), 2 mM glutamine, 100 U/mL penicillin, and 100 μg/mL streptomycin, and kept under standard growth conditions. For the experiments, HT29-MTX cells were seeded on microscopy cover glasses earlier settled into 10-cm^2^ Petri dishes. Confluent cells were gently washed twice with PBS before the bacterial cells were added. *B. bifidum* PRL2010, *B. longum* PRL2022, and *B. breve* PRL2012 were grown as previously described, until a cell density of approximately 5 × 10^7^ CFU·mL^-1^ was reached. The strains were subsequently centrifuged at 3,000 rpm for 8 min, resuspended in PBS (pH 7.3), and incubated with HT29-MTX cell monolayers. After 1 h of incubation at 37 °C, two washes with 2 mL of PBS were used to remove unbound bacteria. The cells were then fixed with 1 mL of methanol and incubated at room temperature for 8 min. Cells were then tinged with 1.5 mL of Giemsa solution (1:20) (Sigma-Aldrich, Milan, Italy) and placed in the dark at room temperature for 30 min. After two washes with 2 mL of PBS, the cover glasses were removed from the Petri dish, placed on a glass slide, and examined using a Zeiss Axiovert 200 phase-contrast microscope (objective, 100×/1.4 oil). Adherent bacteria in 20 randomly chosen microscopic fields were considered for the average calculation. The proportion of bacterial cells that remained attached to the HT29-MTX monolayer was measured to assess the extent of specific host-microbe interaction. The average number of bacterial cells attached to 100 HT29-MTX cells represents the adhesion index^[4,41,42]^. An unpaired Student’s *t*-test was applied to detect statistically significant differences. All analyses were done at least in triplicate.

### RNA extraction and sequencing

Total RNA was isolated as previously described^[43]^. Briefly, cell pellets were resuspended in 1 mL of QIAZOL (Qiagen, United Kingdom) and mixed with 0.8 g of glass beads (106 μm; Sigma) to lyse the cells using a bead beater (2 min shaking, 2 min on ice, repeated). After centrifugation at 12,000 rpm for 15 min, RNA was recovered from the upper phase and purified using the RNeasy minikit (Qiagen, Germany) following the manufacturer’s instructions. RNA quality was assessed using a TapeStation 2200 (Agilent Technologies, USA), and RNA concentration and purity were measured with a spectrophotometer (Eppendorf, Germany). Between 100 ng and 1 μg of total RNA per sample was treated with QIAseq FastSelect - 5S/16S/23S (Qiagen, Germany) to deplete ribosomal RNA. mRNA yield was checked again with the TapeStation 2200. Libraries were prepared using the TruSeq Standard mRNA Library Prep Kit (Illumina, San Diego, CA) and sequenced using a NextSeq 500 platform with a high-output v2.5 kit (150 cycles, single-end), yielding a total of 85,103,471 reads, with an average of 7,091,956 reads per sample. The obtained reads were filtered to eliminate low-quality reads (minimum mean quality, 20; minimum length, 150 bp), as well as any residual ribosomal locus-encompassing reads using the METAnnotatorX2^[44]^. This process retained 54,579,368 high-quality reads, with an average of 4,548,281 reads per sample. Bowtie2 software^[45]^ was used to align the obtained reads to the reference genome of each bifidobacterial strain used. Htseq-counts script of HTSeq software in “union” mode was used for the quantification of reads mapped to individual transcripts^[46]^. Genes with low expression [counts per million (CPM) < 1] were excluded from further analysis. Count data were normalized using the trimmed mean of M values (TMM) method, and differential expression analysis was carried out with the EdgeR package^[47]^, reporting results as log2 fold change (logFC) between experimental and control conditions.

### Statistical analysis

Differences in *B. bifidum* PRL2010 growth in response to melatonin exposure were evaluated by nonparametric independent-samples Kruskal–Wallis test analysis (IBM SPSS Statistics for Windows). The edgeR package was used for the comparison of count-based expression data across different bacterial growth conditions^[47]^. Specifically, raw counts were transformed into CPM and log2-counts per million (log-CPM) values using the CPM function of edgeR. Genes with low counts (CPM < 1) across all conditions were excluded. The TMM method was used to normalize the read counts for variations in library size (sample-specific effect). In the differential gene expression analysis, the log2-ratio was used to represent the average log-fold-change (logFC) in gene expression between the reference sample and each test sample. The false discovery rate (FDR) procedure was used in multiple hypothesis testing to adjust for multiple comparisons.

## RESULTS AND DISCUSSION

### Effects of melatonin on ICST transcriptomes

Given the still unexplored role of melatonin found in human breast milk on the intestinal microbiota of infants, a synthetic microbial community, representing a very common I-CST dominated by bifidobacteria and with the presence of other species (ICST-BI/EN) [Supplementary Table 1]^[39]^, was established using healthy infant-derived bacteria. In detail, after combining the six dominant bacterial species represented by *Bifidobacterium breve*, *B. bifidum*, *Bifidobacterium longum*, *Enterococcus faecalis*, *Enterobacter hormaechei*, and *Escherichia coli* (i.e., abundance > 2%) in physiological proportions within a gut-simulated medium, the model was subsequently exposed to melatonin or maintained in the absence of this hormone (control condition). The strains used for establishing this synthetic microbial community (and representing ICST-BI/EN) were selected based on their genetic and ecological best representation of each species, i.e., designed as reference strains, using the RefBifSelector tool that we recently developed^[48]^. RNA was then extracted and subjected to RNAseq analysis. The latter produced a total of 46,008,027 quality-filtered reads, with an average of 7,668,004 reads per sample [Supplementary Table 2]. In this context, only genes with a log2 fold-change in transcription ≥ 1 and a *P*-value ≤ 0.05, determined by correcting for multiple comparisons using the FDR procedure, were considered to be significantly differentially transcribed between I-CST grown in the presence of melatonin compared with ICST-BI/EN grown in the same medium without this hormone.

**Figure 1:**
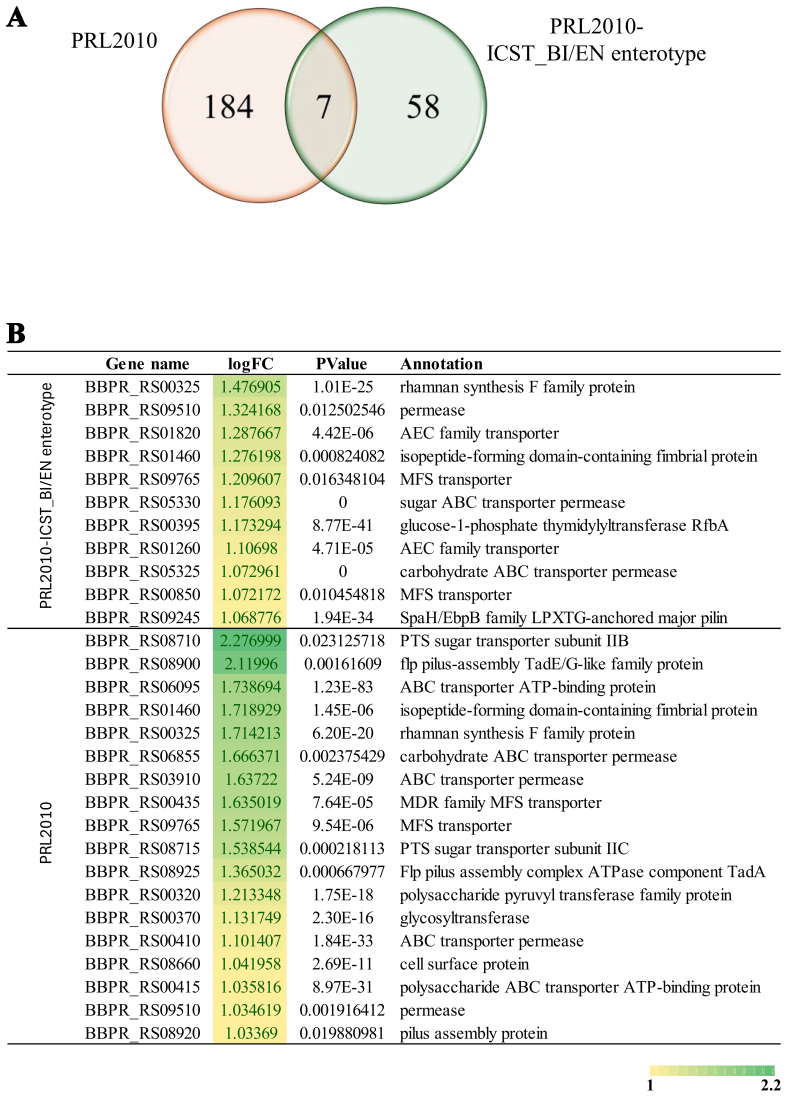
Effects of melatonin on *B. bifidum* PRL2010 transcriptomes. (A) Displays a Venn diagram illustrating the number of shared and unique *B. bifidum* PRL2010 upregulated genes, when exposed alone (on the left) or with other microorganisms representing the ICST-BI/EN (on the right), in the presence of melatonin hormone *vs.* control; (B) Depicts the statistically significantly expressed *B. bifidum* PRL2010 genes (with logFC in transcription ≥ 1 in combination with a *P*-value ≤ 0.05) encoding extracellular structures and transporters possibly related to the interaction with the host. logFC: log2 fold change.

The number of statistically significant upregulated genes was determined to be 58, 22, 15, 13, 11, and 10 for *B. bifidum* PRL2010, *Enterobacter hormaechei* 179F, *Bifidobacterium longum* PRL2022, *Enterococcus faecalis* 2F, *Bifidobacterium breve* PRL2012, and *Escherichia coli* 52F, respectively [Supplementary Table 3]. Since bifidobacteria are known to play an important role in infant health, especially in early gut colonization, we performed a detailed analysis of the functions of the upregulated genes in these species^[17,49]^. Specifically, we categorized the genes upregulated after melatonin exposure using clusters of orthologous groups (COGs). This analysis showed that melatonin increased the expression of *B. bifidum* PRL2010 genes involved in membrane biogenesis and carbohydrate metabolism (COG-M and COG-G). This is in line with findings in plants, where melatonin promotes carbohydrate metabolism and enhances stress tolerance by increasing sugar levels and photosynthesis^[50]^. In mammals, melatonin improves glucose uptake and insulin sensitivity, and protects cardiac tissues by reducing oxidative stress and supporting mitochondrial function. These shared effects across systems highlight melatonin’s conserved role in regulating energy metabolism^[51]^. Interestingly, 11 genes with yet unclear functions were also identified. In addition, analysis of the *B. breve* PRL2012 and *B. longum* PRL2022 transcriptomes showed increased expression of genes predicted to be implicated in carbohydrate metabolism and various unknown functions. Specifically, in both latter bifidobacterial strains, a great part of the upregulated genes encode permeases, suggesting that contact with melatonin may induce an increase in the transcription of transporter-encoding genes that facilitate cellular internalization of specific substances [Supplementary Table 3]. However, the varying number of transcriptionally upregulated genes among the three bifidobacterial strains suggests that melatonin substantially impacts the transcriptome of *B. bifidum* PRL2010 compared with those of the two other bifidobacterial strains. Since *B. bifidum* PRL2010 is considered a highly representative bacterial species of the infant gut microbiota^[48,52]^, we decided to perform an *in-depth* analysis focused on this bifidobacterial strain. Specifically, a functional analysis of the upregulated genes related to carbohydrate transport and metabolism (COG-G) was done. Notably, two upregulated genes, BBPR_RS05325 and BBPR_RS05330 [Figure 1], were expected to be involved in the production of lacto-N-biosidase, an enzyme that releases lacto-N-biose I from lacto-N-tetraose, a key HMO component^[53]^. This finding suggests that melatonin activates HMO metabolism-related genes to favor the bifidobacterial colonization of the ecological niche, i.e., the infant gut, where the predominant carbohydrate sources originate from human milk glycans [Supplementary Table 3]. Additionally, two genes involved in pilus IV biogenesis (BBPR_RS01460 and BBPR_RS09245) and two genes involved in cell envelope-associated exopolysaccharide biosynthesis (BBPR_RS00395 and BBPR_RS00325) were identified [Figure 1 and Supplementary Table 3]. Both these structures are extracellular and have been described as involved in microbe-host interactions^[54,55]^. Cell surface polysaccharides represent a key effector component capable of inducing an immune response, highlighting the important role of these structures in the interactions between bifidobacteria and the host and with other members of the intestinal microbiota. These results suggest that melatonin may exert a strain-specific transcriptional modulation by directly interacting with bacterial cells at the molecular level. The heterogeneous transcriptomic responses observed among bifidobacterial strains indicate a potential for selective signaling or metabolic crosstalk, especially in *B. bifidum* PRL2010, where melatonin seems to activate genes crucial for host colonization and HMO metabolism.

### Adherence of *B. bifidum* PRL2010 to HT29-MTX cells monolayers

**Figure 2:**
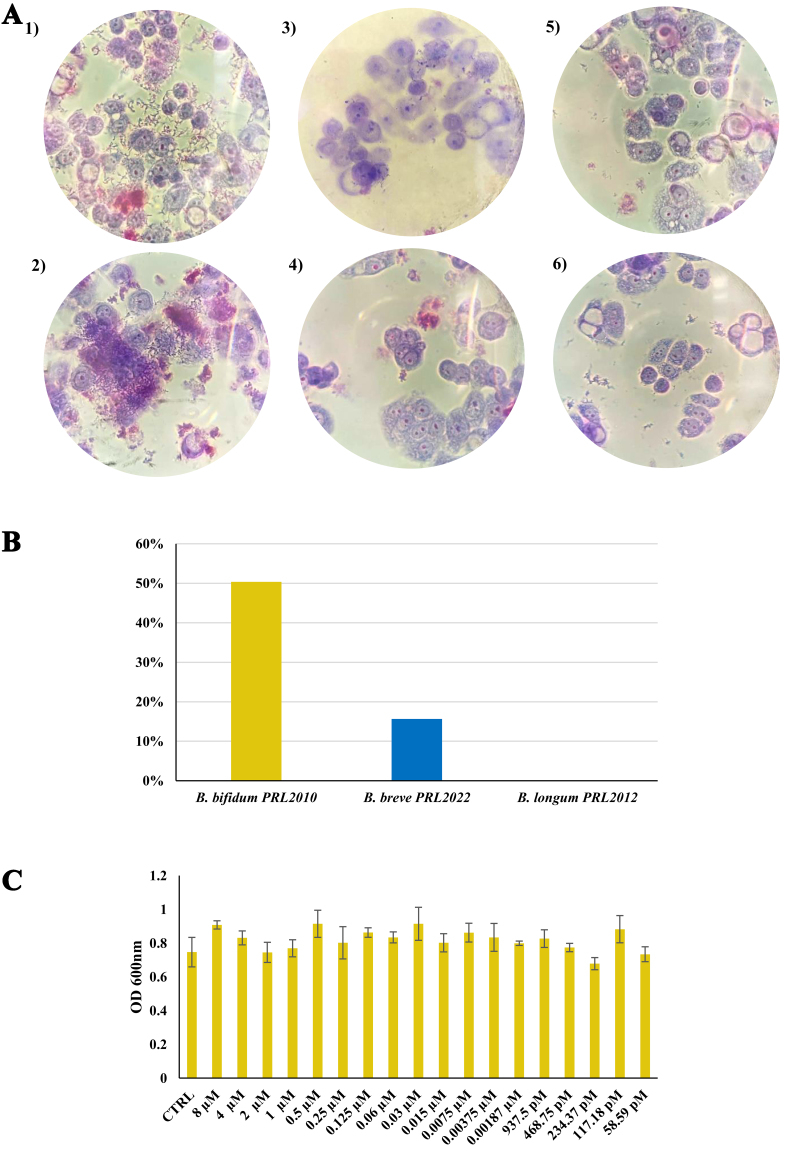
Adherence of *B. bifidum* PRL2010, *B. longum* PRL2022, and *B. breve* PRL2012 to HT29-MTX cells monolayers and growth assay of *B. bifidum* PRL2010 in the presence of different concentrations of melatonin. (A) Displays the light microscopic images of HT29-MTX monolayers as observed with Giemsa staining of bifidobacterial species grown in the absence (1, 3, 5) or in the presence of melatonin (2, 4, 6), respectively. The bifidobacterial species shown in each image are: (1, 2) *B. bifidum* PRL2010, *B. breve* PRL2022 (3, 4), and *B. longum* PRL2012 (5, 6); (B) Depicts the percentage increase in adhesion capacity of *B. bifidum* PRL2010, *B. longum* PRL2022, and *B. breve* PRL2022 when in the presence of melatonin, compared to their adhesion in the absence of the hormone; (C) Represents the growth assay of *B. bifidum* PRL2010 in the presence of different amounts of melatonin. Each pillar shows the bacterial growth at the 19 selected melatonin concentrations from 8 µM to 58.59 pM or under control conditions (CTRL, growth of the bifidobacteria without the hormone). The OD_600nm_ values were expressed as the average of three independent replicates. No statistical significances were exhibited between the conditions, *P*-value > 0.

To corroborate the notion that melatonin influences host colonization by promoting the expression of adhesion-related genes, we investigated the effects of melatonin on the adhesion ability of *B. bifidum* PRL2010, *B. breve* PRL2012, and *B. longum* PRL2022 to a model of human intestinal mucosa. For this objective, the adhesion index was calculated by exposing HT29-MTX monolayers to each strain grown in MRS with and without melatonin, following a protocol previously described^[40,41]^. Interestingly, an increase of 50% in the adhesion index to cell monolayers was observed for *B. bifidum* PRL2010 cells grown in contact with melatonin (adhesion index of 286,000) compared with *B. bifidum* PRL2010 cultivated without melatonin (adhesion index of 190,667) (*t*-test *P*-value < 0.01). The other two strains showed a different trend, since the behaviors observed with or without melatonin were not significantly different. Specifically, *B. longum* PRL2022 cells showed a slight increase (+15%) in the adhesion index in the presence of melatonin compared with *B. longum* PRL2022 grown without melatonin, while *B. breve* PRL2012 does not show any variation with or without melatonin [Figure 2A]. These data show that the presence of melatonin has a more pronounced impact on *B. bifidum* PRL2010 cells compared with other bifidobacterial strains, confirming the transcriptomic data.

### Effects of melatonin on *B. bifidum* PRL2010 growth

We initially assessed whether melatonin influences bifidobacterial growth in the intestinal environment by either promoting or reducing bacterial loads. To this end, *B. bifidum* PRL2010 was cultured in MRS broth supplemented with a wide range of melatonin concentrations (from 58.59 pM to 8 μM), encompassing the physiological levels detected in human breast milk, including variations observed across different individuals, lactation phases, and day–night cycles^[56]^. Interestingly, this growth assay did not reveal any statistically significant differences in the growth performance of the PRL2010 strain with respect to the control (strain grown in the absence of melatonin) (Kruskal-Wallis Test *P*-value > 0.05) [Figure 2]. These findings, therefore, suggest that melatonin neither promotes nor inhibits the growth of *B. bifidum* PRL2010. Since melatonin did not significantly affect bifidobacterial growth at any of the tested concentrations, we selected 2 μM as the standard concentration for all further experiments. This concentration was chosen based on its previous use in similar experimental models^[38]^, allowing us to facilitate comparison with existing literature.

At this point, to assess the molecular impact of melatonin on *B. bifidum* PRL2010, cultures of this strain were exposed to the hormone and their transcriptomes were analyzed. In this context, only genes showing a log2 fold-change in transcription ≥ 1 in combination with a *P*-value ≤ 0.05 calculated through correction for multiple comparisons using the FDR procedure were considered as significantly differentially transcribed between the bifidobacterial strains grown in the presence or in the absence of melatonin. Interestingly, 184 genes were significantly overexpressed in the bifidobacterial strain exposed to melatonin compared with the control [Supplementary Table 4]. It is worth noting that the stimulatory effect of melatonin is more pronounced when *B. bifidum* PRL2010 is cultivated individually rather than within the synthetic microbial community representing the ICST-BI/EN. This observed phenomenon may have different explanations, which will require further investigation. To fully characterize the upregulated genes, a functional categorization analysis of the transcriptomes was performed, revealing genes involved in carbohydrate metabolism and transport (COG-G), cell wall membrane (COG-M), and numerous uncharacterized genes (COG-S). Detailed analysis showed transcriptional upregulation of six genes involved in pilus biogenesis. Four of these genes belong to the Tad pilus locus, with enhanced transcription of *tad*A (the ATPase) and *tad*B, involved in pilus development and assembly, along with the *flp* gene, which encodes the main structural protein of the pilus [Figure 1 and Supplementary Table 4]. Additionally, two genes encoding ABC transporters (BBPR_RS00410 and BBPR_RS00415) were identified [Figure 1]. These findings not only confirm but also build upon previous results from the I-CST, where melatonin was shown to induce the expression of genes encoding extracellular structures and transporters potentially linked to colonization in *B. bifidum* PRL2010. Notably, melatonin is abundant in colostrum^[30]^, suggesting that during early breastfeeding, *B. bifidum* encounters melatonin, which may trigger the upregulation of genes involved in host interaction, such as colonization and adhesion. This interaction could support the persistence of *B. bifidum* in the gut, enhancing its competitiveness and dominance over other strains during the initial stages of colonization of the infant intestine. While additional *in vivo* studies will be needed to confirm these observations, one may hypothesize that reduced exposure to melatonin - for example, in infants who are not breastfed - might impair the colonization efficiency of *B. bifidum*, potentially limiting its beneficial effects. Given the well-documented role of bifidobacteria in supporting immune development and gut homeostasis, these early microbial differences could have downstream implications for infant health^[57-59]^.

This hypothesis fits within the broader framework of evidence highlighting the health benefits of breastfeeding compared to formula feeding^[7,9,60,61]^. In this context, melatonin might represent one of the many bioactive factors in human milk that support the establishment of a beneficial microbiota. Although still speculative, these insights open the door to future studies exploring whether melatonin supplementation in formula could help support the early colonization of key microbial players such as *B. bifidum*.

## CONCLUSION

Melatonin is a hormone secreted by the pineal gland and present mainly in external secretions, such as saliva and breast milk^[27]^. Melatonin in human milk is involved in circadian rhythm, reaching high levels at night to promote sleep^[30]^. In this study, we evaluated possible molecular effects of this hormone on the intestinal microorganisms found in the neonatal guts. Recent studies have suggested bidirectional interactions between the human milk microbiome, particularly bifidobacteria, and the endocrine system^[4]^. However, the mechanisms and implications of these cross-kingdom interactions remain largely unknown. In this context, we decided to focus our analyses on six bacterial species representing a key infant microbial community that is particularly rich in bifidobacteria, which are among the first neonatal intestinal colonizers^[39]^. Functional genomic analyses show that each strain responds differently to melatonin exposure, indicating that the effect of melatonin is species-specific or even strain-dependent. Interestingly, contact with melatonin upregulates several bifidobacterial permeases and some extracellular structures, suggesting that contact with melatonin causes enhanced transcription of genes involved in the uptake of specific substances across the cell membrane. These data are also confirmed by the transcriptomic results of only *B. bifidum* PRL2010 in the presence/absence of melatonin, which represents the most responsive strain impacted by melatonin in the co-cultivation experiments and one of the most representative bacterial species of the neonatal intestinal microbiota^[48,52]^. Furthermore, the potential role of melatonin in promoting bacterial adhesion was investigated through *in vitro* assays using HT29-MTX cells. While these assays offer a useful model to assess bacterial interactions with epithelial cells, they represent a simplified system that cannot fully recapitulate the complexity of the infant intestinal environment. Therefore, although we observed a significant increase in the adhesion capacity of *B. bifidum* PRL2010 following melatonin exposure, further validation in more physiologically relevant systems - such as murine models or clinical studies - is needed to confirm the biological significance of these findings.

Moreover, the synthetic microbial community (ICST-BI/EN) used in this study, while enabling us to control key variables and perform reproducible experiments, represents a strong simplification of the infant gut ecosystem. In particular, it does not fully account for inter-species interactions, host factors, or dynamic environmental changes. Additionally, although transcriptomic analysis provided insights into the molecular responses to melatonin, we were unable to complement these data with metabolomic analyses. Integration of these approaches would have enabled a more complete understanding of the functional consequences of melatonin exposure.

Nonetheless, our results provide a valuable starting point for future investigations into how host-derived molecules like melatonin may influence early-life gut colonization. Such data corroborated previous data documenting the crosstalk between another human milk hormone, insulin, and bifidobacteria^[4]^. Our results provide some first and preliminary insights into possible cross-kingdom interactions between host products like hormones and gut microorganisms, indicating that specific milk hormones trigger species-specific bacterial responses that may influence their ecological fitness with possible functional implications for human health.

## DECLARATIONS

### Availability of data and materials

Raw RNA-seq data are available in the SRA database under accession number PRJNA1214536. Additional raw data supporting the findings of this study are available from the corresponding author upon reasonable request.

### Copyright

© The Author(s) 2025.
